# The effectiveness of targeted therapy for recurrence or metastasis adenoid cystic carcinoma: a systematic review and meta-analysis

**DOI:** 10.1080/07853890.2024.2399867

**Published:** 2024-09-11

**Authors:** Lu Zhang, Hao-Nan Yang, Ying Wang, Dan Li, Zheng Lei, Meng-Qi Yang, Yun-Chang Liu, Jiang He, Yong-Zhong Wu, Jiang-Dong Sui

**Affiliations:** a College of Medicine, Chongqing University, Chongqing, China; b Radiation Oncology Center, Chongqing University Cancer Hospital, Chongqing, China; c College of Bioengineering, Chongqing University, Chongqing, China

**Keywords:** Adenoid cystic carcinoma, targeted therapy, meta-analysis, multiple kinase inhibitors

## Abstract

**Background and purpose:**

Several clinical studies have demonstrated the potential of molecular-targeted agents for the treatment of recurrent or metastatic adenoid cystic carcinoma (R/M ACC). However, there is currently no consensus regarding the efficacy of molecular-targeted agents for patients with R/M ACC. This study aimed to evaluate the therapeutic efficacy and safety of molecular-targeted agents in patients with R/M ACC and provide insights to guide clinical decision-making.

**Materials and methods:**

Five databases (PubMed, Embase, Cochrane, ProQuest, and Scopus) were searched based on the search strategy and selection criteria. Primary endpoints were objective response rate (ORR) and progression-free survival (PFS). The secondary endpoints were disease control rate (DCR), overall survival (OS), metastatic sites, and adverse events (AE). Pooled estimates were calculated using a random-effects meta-analysis.

**Results:**

Finally, 28 studies, involving 849 patients, were included. The most common metastatic sites were the lungs, bones, liver, lymph nodes, and kidneys. The pooled ORR was 4.0% (95% CI, 0.7–8.8%), the pooled DCR was 80.5% (95% CI, 72.2%–87.7%). Compared with other-target drugs, multiple kinase inhibitors (MKIs) improved the ORR (pooled ORR for single-target drugs vs. MKIs: 5.9% vs. 0%). The combination of MKIs and immune checkpoint inhibitors (ICIs) had a significantly higher ORR (17.9% in the axitinib + avelumab group). The pooled median PFS and OS were 8.35 and 25.62 months, respectively. MKIs improved the median PFS compared to other-target drugs (9.43 months vs 5.06 months). In addition, the most common adverse events (AEs) were fatigue (51.6%), hypertension (44.2%), and nausea (40.0%), followed by hand-foot skin syndrome (36.8%), diarrhoea (34.4%), weight loss (34.2%), anorexia (31.8%), rash (31.7%), and headache (29.0%).

**Conclusion:**

The findings of this study suggest that MKIs have a better therapeutic efficacy than single-target drugs in patients with R/M ACC. Future studies are warranted to verify the synergistic role of the combination strategy of MKIs plus ICIs, given the limited number of studies on this topic conducted and published to date.

## Introduction

Adenoid cystic carcinoma (ACC) mostly develops in the salivary glands [1] and accounting for 10–20% of salivary gland tumours [2]. However, nearly 50–60% of these patients will experience a recurrence of disease, and common metastatic sites are the lungs, bones, and liver [3, 4].

Histologically, ACC is categorized by its predominant morphological patterns, including cribriform, tubular, or solid types, which plays a pivotal role in determining the tumour grade [5]. Recent evidences suggested two prognostic ACC molecular subtypes: ACC-I (37%) and ACC-II (63%). ACC-I is featured with overexpression of MYC, and mRNA splicing. Additionally, ACC-I is enriched in NOTCH-activating mutations, leading to a significantly worse prognosis. On the other hand, ACC-II is demonstrated with upregulation of TP63 and receptor tyrosine kinases (AXL, MET, EGFR), showing a less aggressive clinical course [6].

Surgical resection is a top priority for operable ACC patients. Otherwise, inoperable local advanced patients are recommended to receive radiotherapy. For recurrence/metastatic (R/M) ACC, these patients are treated with systemic therapy, expectant management, selected metastasectomy or best supportive care. [7]

The systemic therapy for R/M ACC mainly includes chemotherapy and targeted therapy, but the therapeutic regime remains controversial at present because of the lack of high-quality evidence-based clinical practice. The early studies had shown that the objective response rate of single-agent chemotherapy treating ACC, such as cisplatin [8] and gemcitabine [9], are less than 12%. Notably, the objective response rates for cyclophosphamide-doxorubicin-cisplatin (CAP) [10–13] and cisplatin-vinorelbine [14, 15] range from 18% to 31% but with high incidence of side effects [16]. In the past decade, several clinical trials had explored the potentiality of various targeted drugs in R/M ACC patients. which gradually become the most promising therapeutic regime, including multiple kinase inhibitors (MKIs), NOTCH-targeted therapies, chromatin remodelling agents, agents targeting the PI3K signalling (the phosphatidylinositol-3-kinase (PI3K)/AKT/mammalian target of rapamycin (mTOR) axis), MYB inhibitors, EGFR inhibitors, agents targeting the mouse double minute 2 homolog (MDM2)-p53 axis, and prostate-specific membrane antigen (PSMA)-targeting therapy [17, 18]. However, the curative effect of molecular targeted agents (ranging from 0–15.6%) varies widely among different trials. For example, the ORR and DCR of apatinib (also known as rivoceranib) was 46.2%, 98.5% respectively in Guopei Zhu et al.’ report [19], while only 15.3% and 90.3% in another clinical trial [20], as such, in the TKI treatment of ACC, there is currently no clearly defined optimal choice. Similarly, in various targeted therapies, there is no well-designed trial for head-to-head evaluation of the efficacy and safety of different targeted drugs. Therefore, we sought to performed a meta-analysis to investigate the therapeutic benefit of targeted drugs in R/M ACC patients, aiming to provide a reference for clinical practice.

## Materials and methods

We conducted a systematic literature review and meta-analysis following the Preferred Reporting Items for Systematic Reviews and Meta-analyses (PRISMA) reporting guidelines [21]. This protocol is available online at PROSPERO (CRD42022376157). PROSPERO Registration Number: CRD42022376157; https://www.crd.york.ac.uk/prospero/display_record.php?ID=CRD42022376157

### Search strategy

**Figure 1.**
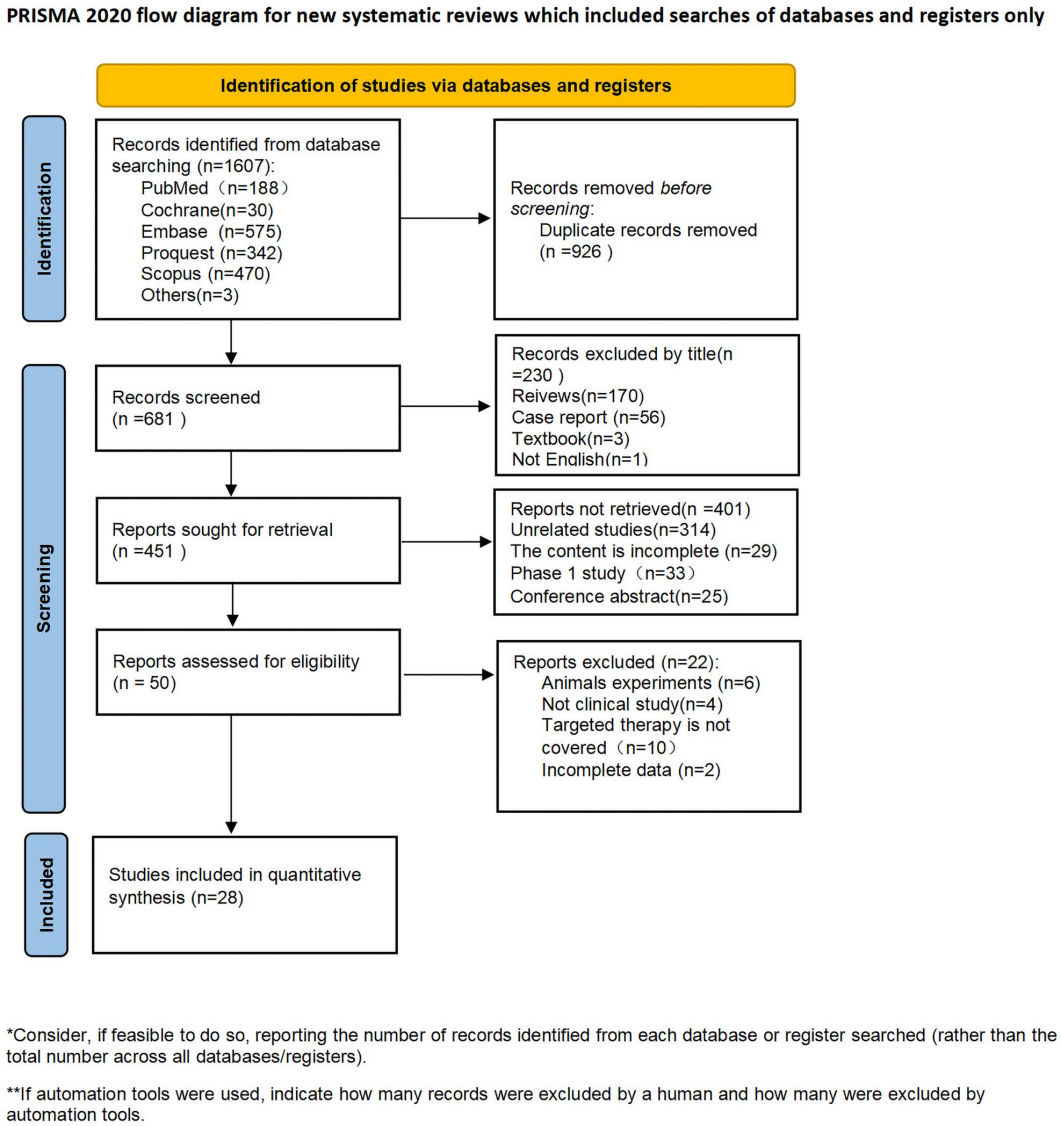
Flow diagram of included and excluded studies.

Two investigators independently searched five databases: PubMed, Embase, Cochrane, ProQuest, and Scopus. The search was performed until November 30, 2022, and the detailed search strategy is provided in the Supplemental Data. The search strategy utilized Medical Subject Heading (MeSH) terms and all synonyms for carcinoma, Adenoid Cystic in combination with MeSH, and all synonyms for molecular-targeted therapy. To ensure maximum sensitivity and to identify all relevant studies, conference abstracts were also considered. A PRISMA flow diagram is shown in Figure 1.

### Study election and data extraction

The inclusion criteria for this study were as follows: (1) patients diagnosed with locally recurrent and/or metastatic adenoid cystic carcinoma. (2) Patients receiving targeted drug therapy as part of their treatment. These patients may also have received other treatments, such as radiotherapy or chemotherapy. (3) Sufficient data available for quantitative meta-analysis, including the reporting of a comparative OR hazard radio for at least one outcome measure such as progression-free survival (PFS), overall survival (OS), disease control rate (DCR), or grade ≥ 3 treatment-related adverse events, were used to select eligible studies for inclusion in the meta-analysis.

The exclusion criteria were as follows: (1) reviews, surveys, letters, case reports, book chapters, and commentary articles; (2) patients who did not receive targeted drug therapy, the content of the study deviated from the aims of this study; (3) studies involving nonhuman subjects; (4) written in a language other than English; (5) literature that did not have original text; (6) literature that did not contain data; (7) studies with fewer than 10 patients; and (8) studies with less than 6 months of follow-up.

### Study election

After conducting the literature search, two investigators eliminated all duplicate studies obtained from different databases. We then independently screened the titles and abstracts of all the remaining articles to exclude studies that did not meet the inclusion criteria. The researchers carefully read the complete literature for possible inclusion and screened those that ultimately met the criteria. Any disagreements regarding study eligibility that remain after discussion can be resolved by consulting another investigator.

By eliminating duplicates and screening for relevance based on the inclusion criteria, investigators can reduce the risk of bias and ensure that their review is based on high-quality studies.

### Data extraction

The following information was extracted from each included study: first author, year of publication, country, age, mean age, study type, invention drags, total number of patients, number of males and females, number of all adverse events, median age, follow-up time, primary end-point, secondary end-point, criteria for response, and criteria for adverse events AEs. The clinical responses included progression-free survival (PFS), overall survival (OS), disease control rate (DCR), objective response rate (ORR), complete response (CR), stable disease (SD), partial response (PR), and progressive disease (PD).

OS was defined as the time between the date of treatment initiation and death for any cause, whereas PFS was defined as the time between treatment initiation and first assessment of progression or death for any cause. CR, complete remission; PR, partial remission; SD, stable disease; and PD, progressive disease; AEs, adverse events. The ORR refers to the objective response rate, which is the sum of the proportions of complete and partial remission. DCR refers to the disease control rate, which is the percentage of cases with remission (PR + CR) and stable disease (SD) after treatment among evaluable cases.

### Assessment of study quality

Two investigators assessed the risk of bias based on the original study using the Newcastle-Ottawa Scale (NOS) for cohort or case-control studies. The included single-arm studies were assessed using the methodological index for non-randomized studies (MINORS) [22]. The JBI Critical Appraisal Checklist for Case Series assesses retrospective studies without a comparison group [23].

### Statistical analysis

A random-effects model was used to pool the single-group rates of DCR and ORR. Given the differences in each study intervention, we conducted a meta-analysis by classifying the included studies into RTK and other treatment subgroups. We used Stata software (version 14.0) for data analyses. Then, we analyzed their heterogeneity and sensitivity, and we show pictures of the funnel plot and sensitivity analysis in the Supplemental Figure.

As most studies did not report detail data about PFS and OS, the survival data was extracted from Kaplan-Meier (K-M) curves using software Engauge Digitizer version 11.3, then survival curve is pooled by ‘MetaSurv’ packages in RStudio (R version 4.3.0) [24, 25].

## Results

### Systematic review and study characteristics

The literature search yield 1607 publications, from 50 eligible articles, 28 studies were included according to the inclusion criteria [19, 20, 26–51]. Most of the studies were single-arm Phase II trials conducted in 849 patients with R/M ACC. The study’s characteristics are listed in Table 1. The effectiveness of multiple kinase inhibitors (MKIs) was reported in 19 studies, EGFR-TKIs in four studies, targeting the PI3K/AKT/mTOR axis (AKT inhibitor and Everolimus) in two studies, chromatin remodelling agents (vorinostat) in one study, mTOR inhibitor (everolimus) in one study, and NOTCH inhibitor (AL101) in one study, MYB inhibitor (ATRA) in one study (Table 2). The median age ranged from 46 (17–67) years to 63 (31–73) years, while the median follow-up time ranged from 4 to 77 months. The most common metastatic sites were the lungs, bones, liver, lymph nodes, and kidneys. Detailed information is provided in Supplemental Table S1.

**Table 1.** Characteristics of included studies.

Study	Country	Study type	Recruitment/case review period	Intervention	N	Median age (range)	M/F	Primary end-points	Secondary end-points	Criteria for response	Criteria for AEs
Hotte 2005[26]	USA	single-arm, Phase II study	2002–2003	Imatinib	16	47(31–69)	6/10	ORR CR PR	DOR	RECIST	CTCAE
Ching-Hung Lin 2005[27]	China	single-arm, Phase II study	NA	Imatinib	5	58(33-–64)	2/3	ORR CR PR	DOR AEs	RECIST	CTCAE
Agulnik 2007[28]	Canada	Phase II study	2004–2006	Lapatinib	20	52(38–72)	12/8	ORR CR PR	SD mPFS OS AEs	RECIST	CTCAE
Locati, LD (2009)[29]	Italy	Phase II study	2004.4-12	Cetuximab	23	45(19–67)	14/9	CBR CR PR SD	NA	RECIST	CTCAE
Ghosal 2011[30]	UK	single-arm Phase II study	2003–2007	Imatinib + cisplatin	28	47(22–74)	12/16	ORR CR PR	AEs	RECIST	CTCAE
Chau 2012[31]	Canada	single-arm Phase II trial	2007–2008	sunitinib	14	60 (40–70)	6/8	ORR CR PR	SD PFS OS AE	RECIST	CTCAE
Hitre 2013[32]	Hungary	single-arm Phase II study	2005–2012	Cetuximab	21	L 57(23–71) M 63(31–73)	L 3/6 M 4/8	PFS	CR、PR、OS、AEs	RECIST	CTCAE
Kim 2014[33]	Republic of Korea	single-arm Phase II study	2008–2010	Everolimus	34	54(27–73)	18/16	PFS	ORR DCR DOR OS AEs	RECIST	CTCAE
Thomson 2014[34]	United Kingdom	single-arm Phase II study	2009–2011	Sorafenib	23	51(36–73)	7/16	PFS	RR CR PR TTP OS AEs	RECIST	CTCAE
Jakob 2015[35]	USA	Phase II study	2004–2007	Gefitinib	18	ACC 50(26–80) non-ACC 59(29–76)	ACC 13/5 non-ACC 10/8	RR CR PR	PFS OS DCR SD AEs	RECIST	CTCAE
Keam 2015[36]	Republic of Korea	single-arm Phase II study	2011–2013	Dovitinib	32	53(23–70)	12/20	PFS	ORR DCR DOR OS AEs	RECIST	CTCAE
Ho,A.L.2015[37]	USA	single-arm Phase II study	NA	MK-2206	16	NA	6/10	ORR	PFS OS AE	RECIST	CTCAE
Ho,A.L.2016[38]	USA	single-arm Phase II trial	2012–2013	Axitinib	33	56(39–78)	18/15	BOR CR PR SD PD	PFS、AEs	RECIST	CTCAE
Ho,A.L.2016[39]	USA	single-arm Phase II study	2018–2022	Regorafenib	38	NA	NA	BOR CR PR	AEs	RECIST	CTCAE
Locati 2016[40]	Italy	Phase II study	2010–2012	Sorafenib	19	50(21-70)	20/17	RR CR PR	ORR PFS DCR OS AEs	RECIST	CTCAE
Guigay, J 2016[41]	France	Phase II study	2013–2015	Pazopanib	49	NA	NA	6-mos PFS rate	CR PR	RECIST	CTCAE
Dillon 2017[42]	America	single-arm Phase II trial	2012–2013	Dovitinib	35	56(28–75)	17/18	ORR TGR CR PR	PFS OS CBR AEs	RECIST	CTCAE
Goncalves 2017[43]	USA	single-arm Phase II study	NA	Vorinostat	30	53(21–73)	11/19	ORR CR PR	PFS OS SD AEs TTR	RECIST	CTCAE
Tchekmedyian 2019[44]	America	single-arm phase II trial	2016–2017	Lenvatinib	32	58(38–73)	12/20	ORR CR PR	PFS AE	RECIST	CTCAE
Locati 2020[45]	Italy	single-arm phase II study	2015–2017	Lenvatinib	28	55 (22–73)	12/16	ORR CR PR	QOL PFS OS DOR AE	RECIST	CTCAE
Ferrarotto, R 2020[46]	USA	single-arm phase II trial	NA	AL101	39	NA	NA	ORR CR PR	AE DOR	RECIST	NA
Kang 2021[47]	Republic of Korea	prospective Phase II study	2016–2017	Axitinib	60	56 (26–77)	28/32	ORR CR PR	OS AE PFS DOR	RECIST	CTCAE
Feeney 2021[48]	UK	single-arm, Phase II study	NA	Lenvatinib	23	46(17–67)	11/12	PFS OS ORR	NA	RECIST	NA
Guopei Zhu 2021[19]	China	prospective Phase II study	2016–2017	Apatinib	68	48.5 (23–71)	25/43	PFS	ORR OS AEs	RECIST	CTCAE
Hanna, GJ 2021[20]	USA	single-arm, Phase II study	2019–2020	ATRA	18	58(40–78)	7/11	ORR CR PR	AE PFS	RECIST	CTCAE
Ferrarotto 2022[50]	America	Phase II study	2019–2022	Axitinib + Avelumab	28	NA	NA	ORR	OS PFS DOR AE	RECIST	CTCAE
Ning Su 2022[51]	China	Retrospective study	2018–2020	Anlotinib	19	48(23–76)	9/10	DCR PFS OS AEs	NA	SYSUCC	CTCAE
Hanna, GJ 2023[20]	USA	single-arm, Phase II study	2020.01-2021.04	apatinib(Rivoceranib)	80	54.5 (28–76)	42/38	ORR DCR	DOR PFS OS TTP AE	RECIST	CTCAE
					849						

N: Number; M/F: Male/Female; L:locally advanced disease; M:metastases; CR: complete response; PR: partial response; SD: stable disease; PFS: progression-free survival; OS: overall survival; AE: adverse events; ORR: objective response rate, sum of CR and PR; DCR: disease control rate,sum of CR, PR and SD; DOR: duration of response; TTP: time to progress; BOR: best overall response; CBR: clinical benefit rate; TGR: tumour growth rate; QOL: quality of life; RECIST: response evaluation criteria of solid tumour; CTCAE: common terminology criteria for adverse events; SYSUCC: sun yat-sen university cancer center; NA: Not available.

**Table 2.** Response rate to targeted therapies of included clinical trials.

Study	Patients(n)	Therapy	Target	ORR	DCR	PFS
MKIs						
Hotte 2005	15	Imatinib	BCR/ABL, v-Abl, PDGFR, c-Kit	0.0%	60%	2.2
Ching-Hung Lin 2005	5	Imatinib	BCR/ABL, v-Abl, PDGFR, c-Kit	0.0%	20.0%	NA
Ghosal 2011	28	Imatinib + cisplatin	BCR/ABL, v-Abl, PDGFR, c-Kit	10.7%	75.0%	15
Chau 2012	13	Sunitinib	VEGFR PDGFR RET FLT3	0.0%	84.60%	7.2
Thomson 2014	19	Sorafenib	VEGFR PDGFR RAF c-Kit FLT-3	10.5%	78.9%	11.3
Keam 2015	32	Dovitinib	VEGFR FGFR FLT3 PDGFR KIT	3.1%	96.9%	6
Ho, A. L.2016	33	Axitinib	VEGFR c-Kit PDGFR	9.1%	84.8%	5.7
Ho, A. L.2016	38	Regorafenib	VEGFR KIT PDGFR, RET, FGFR, RAF	0.0%	44.7%	NA
Locati 2016	19	Sorafenib	VEGFR PDGFR RAF c-Kit FLT-3	10.5%	57.9%	8.9
Guigay, J (2016)	46	Pazopanib	VEGFR, PDGFR, RAF, c-Kit, FGFR, FLT3, ITK, LCK	2.2%	78.3%	5.9
Dillon 2017	34	Dovitinib	VEGFR FGFR KIT PDGFR RET CSF1-R TrkA FLT3	5.9%	70.6%	8.2
Tchekmedyian 2019	32	Lenvatinib	VEGFR FGFR KIT PDGFR RET	15.6%	90.6%	17.5
Locati 2020	26	Lenvatinib	VEGFR FGFR KIT PDGFR RET	11.5%	88.5%	9.1
Kang 2021	27	Axitinib (rivoceranib)	VEGFR KIT PDGFR	0.0%	100.0%	10.8
Feeney 2021	23	Lenvatinib	VEGFR FGFR KIT PDGFR RET	0.0%	47.8%	4.5
Guopei Zhu 2021	65	Apatinib	VEGFR-2	46.2%	98.5%	19.7
Ferrarotto 2022	28	Axitinib + Avelumab	VEGFR KIT PDGFR	17.9%	NA	7.2
Ning Su 2022	19	Anlotinib	VEGFR FGFR KIT PDGFR RET	5.3%	63.2%	10.1
Hanna, GJ (2023)	72	Apatinib (rivoceranib)	VEGFR KIT PDGFR	15.3%	90.3%	9
EGFR Inhibitors						
Agulnik 2007	19	Lapatinib	EGFR HER-2	0.0%	78.9%	3.4
Locati, LD (2009)	23	Cetuximab	EGFR	0.0%	87.0%	6
Hitre 2013	21	Cetuximab + chemotherapy	EGFR	42.9%	76.2%	NA
Jakob 2015	17	Gefitinib	EGFR	0.0%	82.4%	4.3
Targeting the PI3K/AKT/mTOR Axis						
Kim 2014	34	Everolimus	mTOR	0.0%	79.4%	11.2
Ho, A. L.2015	14	AKT inhibitor (MK-2206)	Akt	0.0%	92.9%	9.2
Chromatin Remodeling Agents						
Goncalves 2017	30	Vorinostat	HDACi	6.7%	96.7%	11.4
NOTCH Inhibitors						
Ferrarotto, R (2020)	39	AL101	NOTCH	15.4%	53.8%	NA
MYB Inhibitors						
Hanna, GJ (2021)	18	ATRA	MYB-NFIB	0.0%	61.1%	3.2

MKI: multiple kinase inhibitors; VEGFR: vascular endothelial growth factor receptor; PDGFR: Platelet-derived growth factor receptor; EGFR: epidermal growth factor receptor; PI3K: phosphatidylinositol-3-kinase; mTOR: mammalian target of rapamycin; ORR: sum of complete response (CR) and partial response (PR); DCR: sum of CR, PR and stable disease (SD); NA: not available.

### Quality assessment

The quality of the eight studies was assessed using the Newcastle-Ottawa Quality Assessment Form for Cohort Studies (NOS), with scores ranging from 6 to 8. Twenty single-arm studies were assessed using the MINORS index score ranging from 13 to 15 points, which was acceptable for the present meta-analysis. One retrospective study without comparison was included after it was assessed using the JBI Critical Appraisal Checklist for Case Series. The details of the quality assessments are shown in Table 3.

**Table 3.** Quality assessment of included studies.

A. The Newcastle-Ottawa Scale(NOS) for Cohort study											
Study	Selection			Comparability				Outcome			Total
Agulnik 2007	4			1				3			8
Locati 2009	3			0				3			6
Jakob 2015	4			0				3			7
Locati 2016	4			1				3			7
Guigay 2016	3			0				3			6
Kang 2021	4			2				3			7
Guopei Zhu 2021	3			0				3			6
Ferrarotto 2022	3			0				3			6
B. MINORS index for included non-randomized studies											
Study	I	II	III	IV		Ⅴ	Ⅵ		Ⅶ	Ⅷ	Total
Hotte 2005	2	2	2	2		0	2		2	2	14
Ching-Hung 2005	2	2	2	2		0	2		2	2	14
Ghosal 2011	2	2	2	2		0	2		1	2	13
Chau 2012	2	2	2	2		0	2		2	2	14
Hitre 2013	2	2	2	2		0	2		2	2	14
Kim 2014	2	2	2	2		0	2		2	2	14
Thomson 2014	2	2	2	2		0	2		2	2	14
Keam 2015	2	2	2	2		0	2		2	2	14
Ho,A.L. 2015	2	2	1	2		0	2		2	2	13
Ho,A.L. 2016	2	2	2	2		0	2		2	2	14
Ho,A.L. 2016	2	2	2	2		0	2		2	2	14
Dillon 2017	2	2	2	2		0	2		2	2	14
Goncalves 2017	2	2	2	2		0	2		2	2	14
Tchekmedyian 2019	2	2	2	2		0	2		2	2	14
Locati 2020	2	2	2	2		0	2		2	2	14
Ferrarotto 2020	2	2	2	2		0	2		2	2	14
Feeney 2021	2	2	2	2		1	2		2	2	15
Hanna, GJ 2021	2	2	2	2		0	2		2	2	14
Hanna, GJ 2023	2	2	2	2		1	2		2	2	15
C. JBI Critical Appraisal Checklist for Case Series for included retrospective studies											
Study	Q1	Q2	Q3	Q4	Q5	Q6	Q7	Q8	Q9	Q10	Overall appraisal
Ning Su 2022	Yes	Yes	Yes	Unclear	Yes	Yes	Yes	Yes	Yes	Yes	Include

### Evaluation of efficacy outcomes

Of the 28 studies, three studies used multiple drugs and 25 used single drugs. We divided the single-drug group into six groups, three of which had only one study, which we tabulated separately to analyze ORR and DCR, and the other three were divided into MKI, EGFR inhibitor, and targeting the PI3K/AKT/mTOR axis, and analyzed the DCR and ORR separately for these three groups. We also analyzed DCR and ORR separately for the other three multi-drug studies.

**Figure 2.**
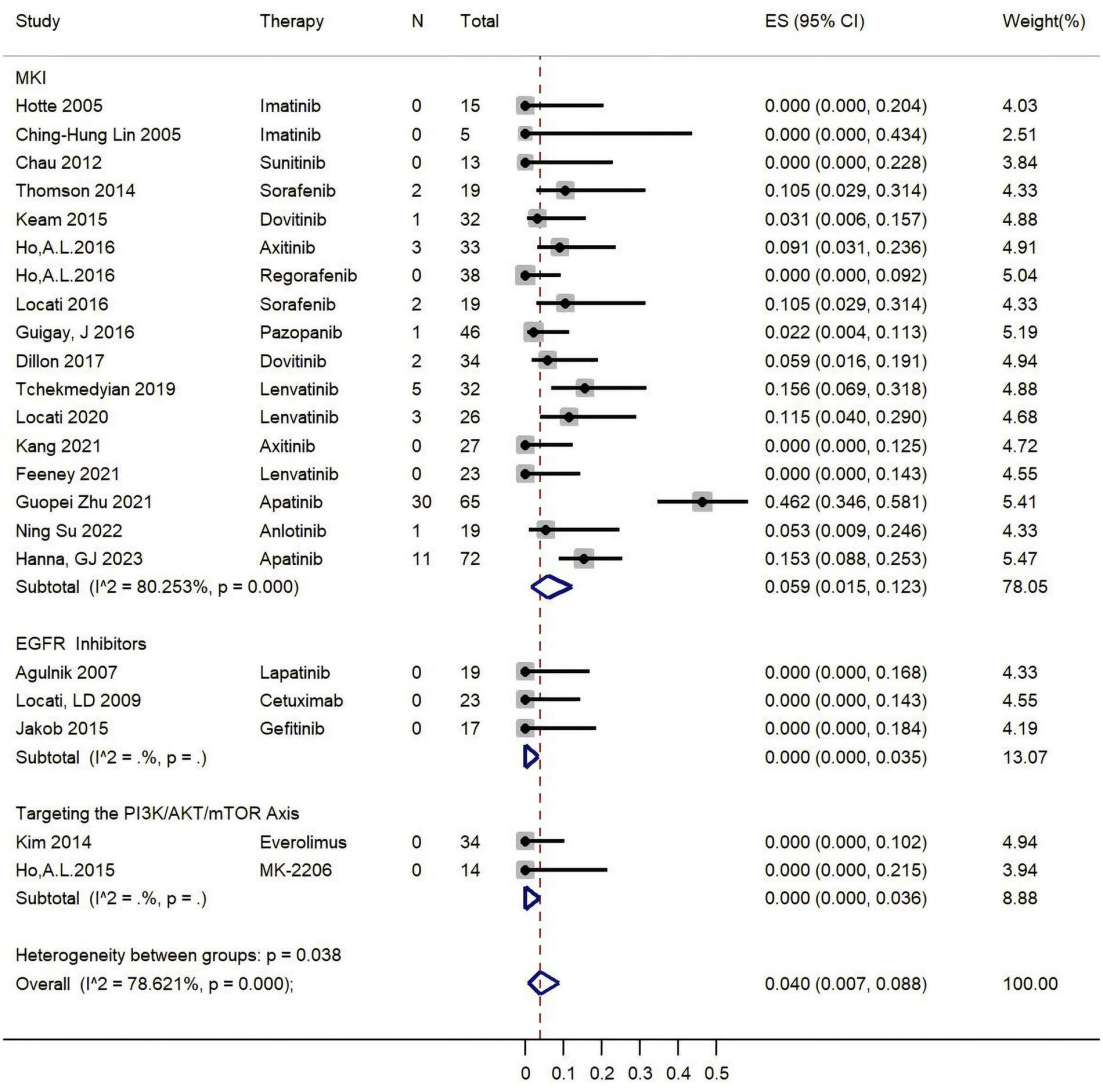
Pooled results of ORR represented by Forest plots. The large diamond at the bottle of the plot represents the pooled rate of all studies. The width of the diamond represents with 95% CI.

As shown in Figure 2, the pooled ORR was 4.0% (95% CI, 0.7%–8.8%). In the subgroup analysis, the pooled ORR was higher in the group of MKIs (5.9%, 95% CI, 1.5%–12.3%) than in the other two groups of single-target drugs (0%, 95% CI, 0–3.5%). The forest plot indicated that MKIs of Apatinib (46.2% ORR) and NOTCH inhibitors (15.4% ORR) were potential candidates, but the first-generation EGFR inhibitors, including Gefitinib and Lapatinib, might not be efficient when treating patients with R/M ACC. The combination of EGFR and chemotherapy also had a potential synergistic effect, with an ORR of 42.9% for cetuximab combined with chemotherapy (Supplement Table S2) [32]. In addition, the combination of immune checkpoint inhibitors (ICIs) and MKIs had a potential synergistic effect; for instance, axitinib combined with avelumab reached a 17.9% ORR (Supplement Table S2).

**Figure 3.**
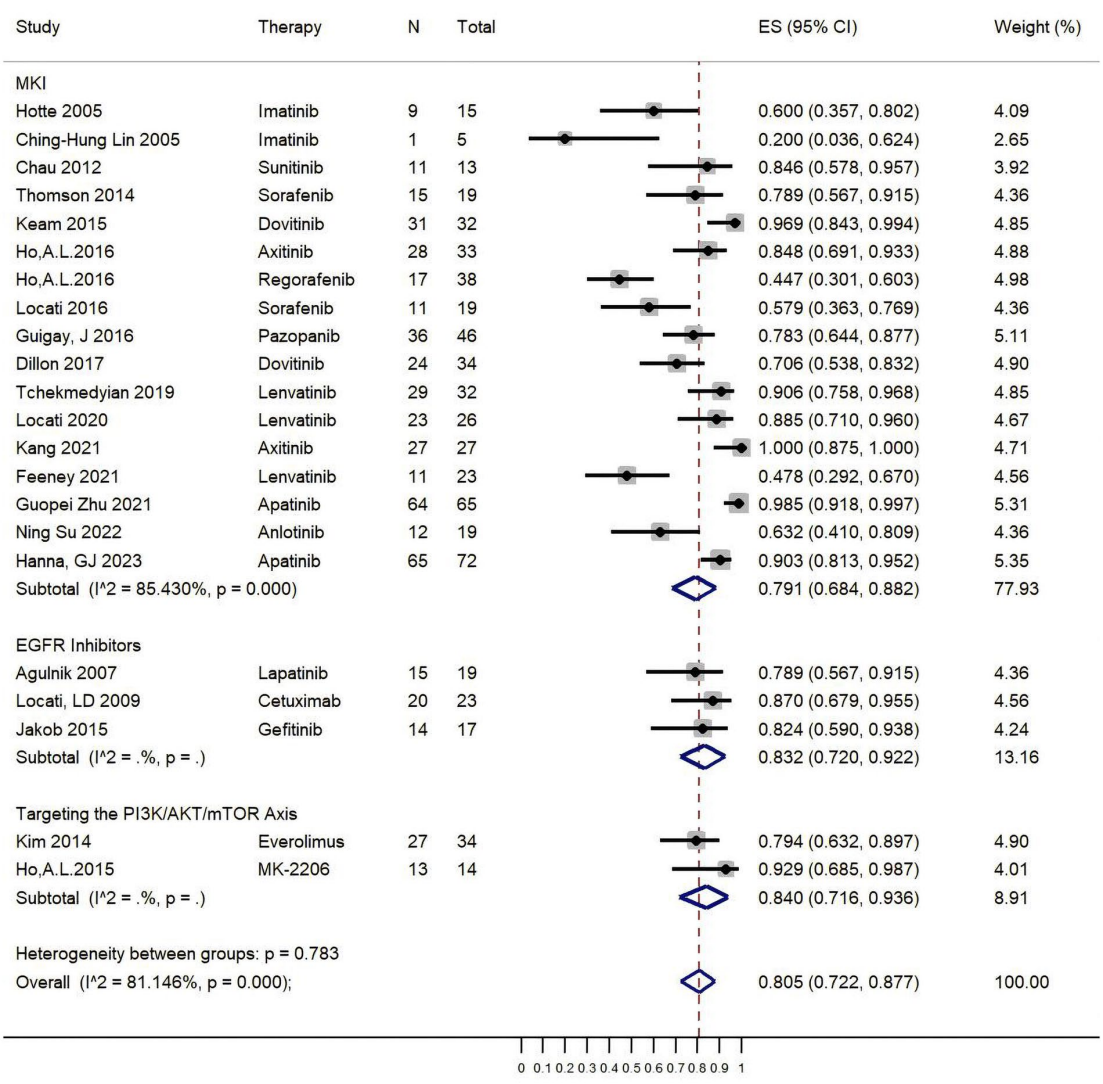
Pooled results of DCR by Forest plots. The large diamond at the bottle of the plot represents the pooled rate of all studies. The width of the diamond represents with 95% CI.

Figure 3 shown that the pooled DCR was 80.5% (95% CI, 72.2%–87.7%). In the subgroup analysis, there were no significant difference between the group of MKIs [79.1% (95% CI, 68.4%–88.2%)], EGFR inhibitors [83.2% (95% CI, 72.0%–92.2%)]. and targeting the PI3K/AKT/mTOR axis [84.0% (95% CI, 71.6%–93.6%)]. The resultant forest plot found that Apatinib has highest DCR up to 98.5%, but Imatinib reached lowest DCR at 20.0%. Chemotherapeutic agents combined with targeted therapy did not enhance DCR (Supplement Table S2).

### Survival benefits of targeted therapy

**Figure 4.**
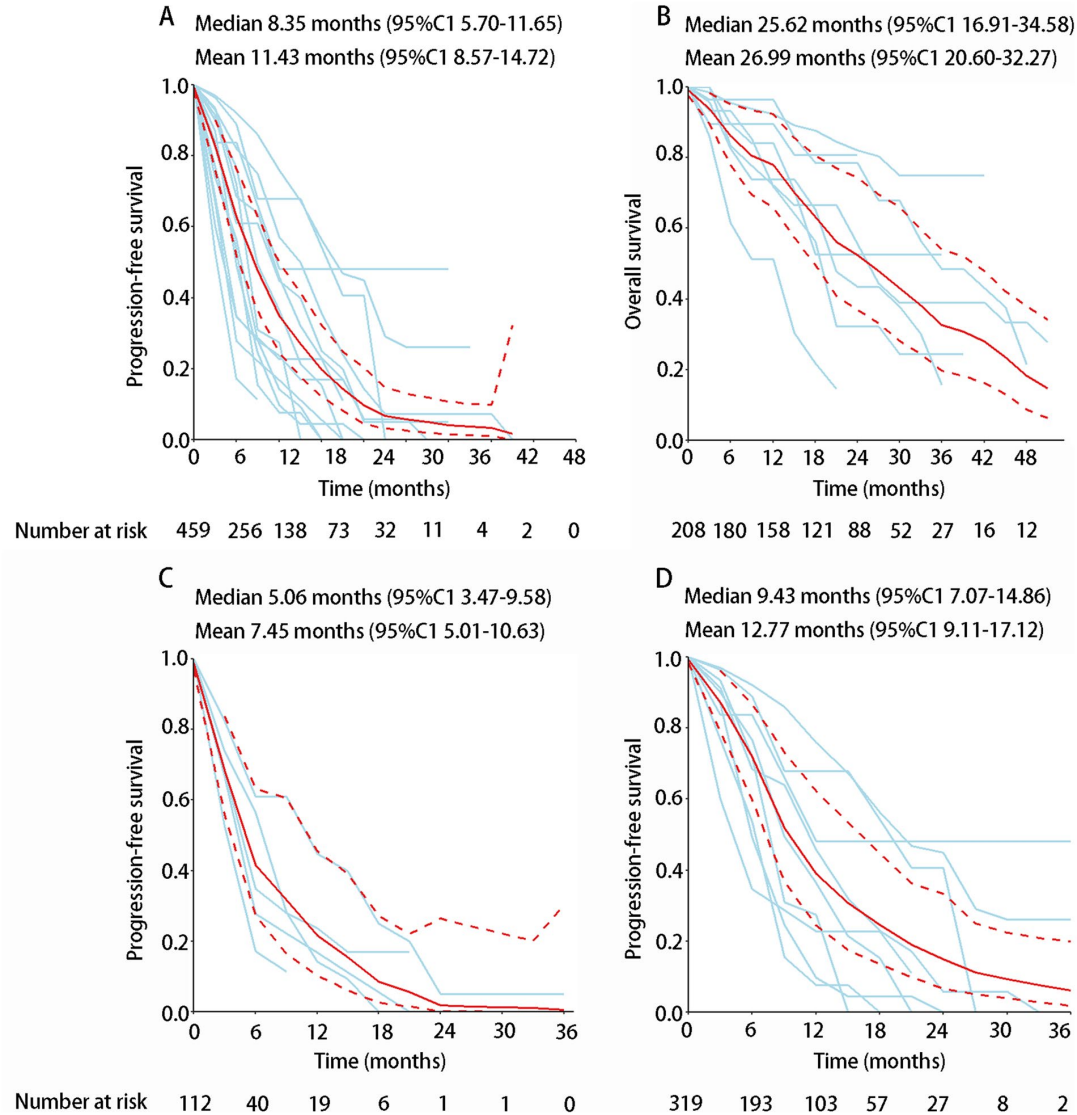
Pooled Kaplan-Meier survival curves of ACC treated with targeted therapy. (A) Pooled Kaplan-Meier PFS curves of ACC targeted therapy; (B) Pooled Kaplan-Meier OS curves of ACC targeted therapy; (C) Pooled summary progression-free survival curves of ACC treated with single-target targeted therapies; (D) Pooled summary progression-free survival curves of ACC treated with MKI targeted therapies. Blue lines represent survival curves for individual studies. Solid red lines represent summary survival curves with 95% CIs (dashed red lines).

To better estimate the PFS of R/M ACC patients treated with targeted therapy, we constructed pooled summary survival curves from data available in the literature. The PFS K-M curves were reported in 15 studies [19, 20, 28–31, 33–36, 38, 44, 48, 49, 51]; the pooled median PFS was 8.35 months (95% CI 5.70–11.65), the 6-month PFS was 60.9% (95%CI 51.9%–76.2%), and the 12- and 24-months PFS was 34.9%(95CI 24.5%–49.8%) and 9.6%(95%CI 4.5%–20.3%) (Figure 4A), respectively. We further found that the pooled median PFS was better in the group of MKIs (9.43 months, 95%CI 7.07–14.86) than the group of single-target drugs (5.06 months, 95%CI 3.47–9.58) (Figure 4C and 4D). In addition, eight studies had completed OS K-M curves [19, 28, 30, 31, 34, 35, 48, 51], and the median OS for ACC patients treated with targeted therapy was 25.62 months (95% CI 16.91–34.58), 6-month OS was 86.1% (95%CI 77.9%–95.2%), and 12- and 24-months OS was 78.0%(95CI 66.0%–92.3%) and 52.4%(95%CI 36.9%–74.4%), respectively (Figure 4B).

### Safety of targeted therapy

Among the 15 studies that reported adverse events, the most common all-grade adverse events (AEs) were fatigue (51.6%; 95% CI, 32.2%–70.7%), hypertension (44.2%; 95% CI, 22.7%–66.9%), and nausea (40.0%; 95% CI, 22.7%–58.5%), followed by hand-foot skin syndrome (36.8%; 95% CI, 19.2%–56.3%), diarrhoea (34.4%; 95% CI, 18.6%–52.0%), weight loss (34.2%; 95% CI, 16.4%–54.8%), anorexia (31.8%; 95% CI, 20.5%–44.2%), and rash (31.7%; 95% CI, 11.2%–56.3%) (Table 4). For grade 3 or higher AEs, rash (19.2%; 95% CI, 2.7%–43.8%) was the most common, followed by hypertension (17.9%; 95% CI, 7.5%–31.1%) and fatigue (14.0%; 95% CI, 6.9%–22.8%). The high incidence of adverse events during NOTCH inhibitor treatment were diarrhoea (78%, grade3 10%), fatigue (67%, grade3 6%), and nausea (60%, grade3 7%) [46]. The most common adverse events to chromatin remodelling agents were fatigue (70%, grade3 10%), lymphopenia (56.7%, grade3 23.3%) and hypertension (50%, grade3 10%) [43]. The most common adverse effects of MYB inhibitors were dry skin (78%, grade3 11.1%), headache (72%, grade3 22.2%), and elevated cholesterol (17%, grade3 0) [49] (Table 5). Statistics on the number of common grade 3 or higher adverse events are presented in Supplement Table S3.

**Table 4.** Incidence rates of the most common adverse events across all grades and grade 3 or higher.

AEs	Any grade		≥Grade 3	
	ES, % (95% CI)	I2, %	ES, % (95%CI)	I2, %
Fatigue	51.6(32.2–70.7)	92.9	14.0(6.9–22.8)	79.2
Hypertension	44.2(22.7–66.9)	93.58	17.9(7.5–31.1)	85.1
Nausea	40.0(22.7–58.5)	90.1	3.2(0.6–7.2)	–
Hand-foot skin syndrome	36.8(19.2–56.3)	87.9	5.5(1.6–10.9)	30.0
Vomiting	35.2(17.6–54.9)	84.8	4.8(0.8–22.7)	–
Diarrhea	34.4(18.6–52.0)	91.9	3.9(1.2–7.5)	31.3
weight loss	34.2(16.4–54.8)	90.3	4.9(1.2–10.2)	48.5
Anorexia	31.8(20.5–44.2)	75.3	5.7(0.9–13.2)	51.4
Rash	29.4(12.1–50.1)	92.6	19.2(2.7–43.8)	84.0
Headache	29.0(14.8–45.4)	86.0	10.7(3.0–21.5)	13.9

**Table 5.** The summary of adverse events of included studies.

Study	Therapy	Classification of Targeted Therapies	N	Fatigue	Hypertension	Nausea	Hand–foot syndrome	Diarrhea	Anorexia	Rash	Headache	Anemia	Thrombocytopenia
Hotte 2005	Imatinib	MKI	16	14(87.5%)	NA	14(87.5%)	NA	10(62.5)	9(56.3)	NA	8(50%)	NA	NA
Ching-Hung Lin2005	Imatinib	MKI	5	2(40%)	NA	4(80%)	NA	1(20%)	3(60%)	1(20%)	NA	2(40%)	1(20%)
Thomson 2014	Sorafenib	MKI	23	22(95.7%)	3(13%)	15(65.2%)	17(73.9%)	18(78.3%)	11(47.8%)	17(73.9%)	NA	1(4.3%)	NA
Keam 2015	Dovitinib	MKI	32	NA	2(6.3%)	17(53.1%)	NA	17(53.1%)	12(37.5)	14(43.8%)	5(15.6%)	3(9.4%)	2(6.3%)
Ho, A. L. 2016	Axitinib	MKI	33	31(93.9%)	32(97%)	15(45.5%)	17(51.5%)	18(54.5%)	17(51.5%)	6(18.2%)	4(12.1%)	6(18.2%)	9(27.3%)
Locati2020	Lenvatinib	MKI	28	NA	22(78.6%)	6(21.4%)	8(28.6%)	12(42.9%)	NA	2(7.1%)	NA	4(14.3%)	3(10.7%)
Kang 2021	Axitinib	MKI	60	11(18.3%)	14(23.3%)	4(6.7%)	6(10%)	7(11.7%)	11(18.3%)	2(3.3%)	5(8.3%)	NA	NA
Guopei Zhu 2021	Apatinib	MKI	68	16(23.5%)	44(64.7%)	NA	27(39.7%)	1(1.5%)	11(16.2%)	NA	NA	5(7.4%)	5(7.4%)
Ferrarotto 2022	Axitinib + Avelumab	MKI	28	17(62%)	9(32%)	NA	NA	8(29%)	NA	NA	NA	NA	NA
Ning Su 2022	Anlotinib	MKI	19	4(21.1%)	6(31.6%)	NA	5(26.3%)	NA	4(21.1%)	NA	NA	NA	NA
Hanna, GJ2023	Apatinib	MKI	80	47 (58.8%)	52 (65.0%)	40 (50.0%)	NA	26 (32.5%)	NA	11 (13.8%)	29 (36.3%)	7 (8.8)%	NA
Hitre 2013	Cetuximab	EGFR Inhibitors	21	5(23.8%)	NA	NA	NA	4(19%)	NA	20(95.2%)	NA	NA	NA
Kim 2014	Everolimus	Agents Targeting PI3K Signaling	34	NA	NA	5(14.7%)	NA	NA	5(14.7%)	10(29.4%)	NA	22(64.7%)	4(11.8%)
Goncalves 2017	Vorinostat	Chromatin Remodeling Agents	30	21(70%)	15(50%)	NA	NA	NA	NA	NA	9(30%)	NA	NA
Hanna2021	ATRA	MYB Inhibitors	18	2(11.1%)	NA	2(11.1%)	NA	NA	NA	2(11.1%)	13(72.2%)	NA	NA

MKIs: Multiple Kinase Inhibitors; EGFR: Epidermal growth factor receptor; PI3K: phosphatidylinositol-3-kinase; NA: not available.

Of the 15 studies that involved all adverse reactions, 11 were MKIs. We concluded that the top all-grade adverse reactions in MKIs were Fatigue (52.5%; 95% CI, 30.2%–74.3%), hypertension (46.1%; 95% CI, 24.9%–67.9%), nausea (43.4%; 95% CI, 24.4–63.4), Hand-foot syndrome (36.8%, 95% CI, 19.2–56.3%), diarrhoea (36.1%; 95% CI, 18.8%–55.2%), anorexia (34.6%; 95% CI, 22.1%–48.3%), weight loss, (34.3%; 95% CI, 16.4%–54.8%) and headache (29.0%; 95% CI, 12.8%–48.3%). At present, it is not possible to identify other-target therapies adverse events (Supplement Table S4).

### Sensitivity analysis and publication bias

We conducted a sensitivity analysis of 28 articles and obtained the following results: *p* = 0.11 (95%CI) (0.04, 0.18). This indicates that none of the articles would affect the meta-analysis results, which means that our study was very stable (Supplementary Figures S1 and S2).

Egger’s and Begg’s tests were performed to evaluate publication bias. Funnel plot the pooled DCR in R/M ACC. P value for Egger: 0.137, P value for Begg:0.257 (Supplementary Figure S3). The test results of the pooled ORR did not show significant publication bias among the included studies (*p* = 0.02 for Egger’s test and *p* = 0.593 for Begg’s test) (Supplementary Figure S4).

## Discussion

Adenoid cystic carcinoma is considered as a slow growing tumour with relatively high 5-year survival rate (90.3%). Nonetheless, nearly 60% patients suffer a recurrence after initial treatment. The optimized treatment of R/M ACC is remained to be explored. This systematic review and meta-analysis showed that the pooled ORR of R/M ACC patients receiving any targeted therapy was 4%. The subgroup analysis indicated that MKIs was better for patients with R/M ACC when comparing with other-target drugs (ORR, 5.9% vs 0%).

This meta-analysis found that MKIs, especially apatinib in the largest cohort study [19], were significantly better in terms of ORR and median PFS than other-target drugs in patients with R/M ACC. However, another clinical trial involving apatinib (now rivoceranib) has reported a modest ORR (15.3%) [20]. The objective response rate (ORR) for recurrent/metastatic ACC after treatment with MKI mostly ranged from 0% to 15.6%. However, the addition of MKIs barely improved the DCR compared with other-target drugs. Hence, there is currently no tyrosine kinase inhibitor (TKI) that distinctly stands out as the preferred choice for ACC.

MKIs (except imatinib) block VEGF-VEGFR signalling and inhibit VEGF-mediated endothelial cell migration and proliferation. Reports have shown that VEGF is overexpressed in ACC compared to benign salivary gland tumours with low VEGF expression levels [52, 53]. This partially explains the higher ORR of MKIs to single-target drugs and indicates the importance of targeting VEGF-VEGFR signalling. Imatinib targets BCR-ABL, PGFRA, and C-Kit and has revolutionized the management of chronic myeloid leukaemia and gastrointestinal stromal tumours, but failed to improve ORR and DCR in patients with R/M ACC [54, 55]. In the study conducted by Kang et al. in 2021 [47], there is a lower response rate of axitinib, but 3 patients crossed over to the experimental group, resulting in a response rate of 11.5%, Median PFS in the axitinib arm was 10.8 months. As compared to the previous study with a single-arm design, Kang et al.’s study had a sufficient sample size and excluded patients prior exposure to anti-angiogenic drugs. Therefore, Kang et al.’s study is more convincing. Furthermore, the treatment with single agent targeting the cKIT, EGFR Fibroblast Growth Factor Receptor (FGFR), and Protein Kinase B (AKT) pathways did not significantly improve clinical outcome of patients with R/M ACC [56]. More efforts are required to identify the therapeutic target for ACC.

EGFR is commonly overexpressed in ACC, making it a potential therapeutic target [57]. In this study, no response was observed in phase II trials testing the first-generation EGFR inhibitors Gefitinib and Lapatinib. One possible reason is that EGFR gene mutations only account for approximately 10% of ACC patients, although the expression of the EGFR protein is common [58, 59]. Moreover, targeting EGFR led to the accumulation of stem-like cells in ACC, which contributed to tumour development [60]. Based on these studies, EGFR inhibitors may not be appropriate for patients with R/M ACC.

Recent studies found that ACC tumours could be categorized into two main subtypes, in which ACC-I exhibited MYC overexpression and NOTCH mutation with poor prognosis, and ACC-II exhibited P63 overexpression and RTK upregulation with improved prognosis [6, 61]. Consistent with Ferrarotto et al.’s report (2022) [6], the gene expression analysis of ACC revealed identified potential therapeutic targets including G2-M checkpoint genes, E2F, MYC targets, IFN, MTORC1, RAS signalling and the FGF/IGF/PI3K and NOTCH pathways [62]. Beyond the good prognosis for primary ACC patients, the newly recognized subtype with enrichment of NOTCH1 activating mutations results in a short median OS of 3.4 years [6, 17]. Thus, targeting NOTCH signalling may improve the outcome of patients with ACC-I. AL101 is a γ-secretase inhibitor that inhibited ACC xenograft growth in a preclinical study. Early results from a phase 2 trial investigating AL101 (NCT03691207) showed promising ORR in patients with R/M ACC. Overall, the molecular classification of R/M ACC should be further investigated in the future to guide the cinical decisions regarding specific targeted agents.

Combination therapy with targeted therapy and chemotherapy or immunotherapy is promising. Preliminary studies, such as those by Savarese et al. [63], showed that combining drugs like vorinostat or olaparib with standard chemotherapy agents like cisplatin and doxorubicin was more effective than monotherapy. Ongoing clinical trials are exploring the efficacy of such combinations in improving patient survival rates and treatment effects, particularly in R/M ACC, with early results indicating synergistic effects and good tolerability [32]. Currently, due to the small sample size in clinical studies, it is challenging to identify subgroups that benefit from them, highlighting the need for effective predictive biomarkers. Further research is needed to understand the biological basis of immune checkpoint inhibitor resistance and to develop more effective treatment strategies, especially considering the diversity of MKIs.

Similar to other reports about adverse events of targeted therapy, skin changes, including rash (29.4%) and hand-foot syndrome (36.8%), were common according to the pooled data. In addition, we found a high pooled incidence of hypertension (44.2%), partially due to treatment with VEFGR inhibitors, such as lenvatinib, apatinib, axitinib, regorafinib, and anlotilib. Other common adverse events, including nausea, anorexia, headache, anaemia, and thrombocytopenia, were comparable to the incidence of targeted therapy in other cancers. Specifically, fatigue (52.5%), hypertension (46.1%), and nausea (43.4%) are the most common adverse events of patients receiving MKIs, while diarrhoea was associated with NOTCH inhibitors. In brief, there were no additional adverse events in patients with R/M ACC receiving targeted therapy when compared to other cancers [64, 65].

Although only approximately 10% of patients with ACC have distant metastasis at diagnosis, more than half of the patients experience distant metastasis when they have recurrence. According to an early study, the lung was the most common site of metastasis, with an incidence of approximately 90%, followed by the bone and brain. The pooled data from the 16 studies that reported metastasis sites were consistent with earlier findings. The pooled incidences of lung and bone metastases were 81.6% and 25.8%, respectively. In contrast to other reports, the incidence of brain metastasis was 4.8%. Moreover, the liver and kidney should also be assessed carefully, with incidences of 23.7% and 9.7%, respectively.

This study had several limitations. First, the heterogeneity was high in the pooled and subgroup analyses. Since all studies included were non-randomized phase II clinical trials, heterogeneity in the design of each trial was inevitable. Furthermore, baseline characteristics, including prior treatment, tumour burden, and ethnicity, also led to high heterogeneity. Second, the pooled outcome estimates of this study were based on single-arm results, which might contain systematic bias and should be interpreted with caution when compared to other results. Third, owing to limited data availability, this study was unable to analyze the relationship between gene mutations in R/M ACC and the efficacy of targeted therapy.

## Conclusion

Currently, the treatment of R/M ACC remains a challenge for both patients and physicians because it is refractory to common systematic treatments. Although no agent has been accepted for R/M ACC treatment, advances in molecular targeted therapy, including MKIs (especially VEGFR inhibitors) and NOTCH inhibitors, may improve outcomes. The combination strategy of molecular-targeted therapy with widely studied immunotherapy should be investigated in the future. Moreover, understanding the molecular classification of R/M ACC is important for building personalized molecular targeted treatments, and more research is needed to achieve this goal.

## Supplementary Material

Supplemental Material

## Data Availability

The original contributions of the study are included in the article/supplementary material. Further inquiries can be directed to the corresponding author.
