# Supplementary material for: The effectiveness of targeted therapy for recurrence or metastasis adenoid cystic carcinoma: a systematic review and meta-analysis
Source: Ann Med. 2024 Sep 11;56(1):2399867. doi: 10.1080/07853890.2024.2399867 (PMC11391875; doi:10.1080/07853890.2024.2399867)
Supplement: Supplemental Material [file IANN_A_2399867_SM5405.zip › suppl_data/Supplemental table_revised - IANN-2023-2501.R2.docx]

Supplementary Table

**Table S1 The most common Metastatic sites**

| Metastatic | ES, % (95% CI) | I2, % |
| --- | --- | --- |
| Lung | 81.6（69.8-91.2） | 83.29 |
| Bone | 25.8（19.6-32.5） | 33.59 |
| Liver | 23.7（14.9-33.7） | 71.27 |
| Nodes | 15.3（9.4-22.2） | 40.54 |
| Kidney | 9.7（5.8-14.3） | 0 |
| Brain | 4.8（1.2-10.1） | 21.74 |
| Skin | 4.2（1.0-9.0） | 0 |

**Table S2 The ORR&DCR of combination therapies**

|  | Study | Therapy | N | Total | ES (95%CI) |
| --- | --- | --- | --- | --- | --- |
| ORR | Ghosal 2011 | Imatinib +cisplatin | 3 | 28 | 0.107（0.037-0.272） |
|  | Ferrarotto 2022 | Axitinib + Avelumab | 5 | 28 | 0.179（0.079-0.356） |
|  | Hitre 2013 | Cetuximab +chemotherapy | 9 | 21 | 0.429(0.245-0.635) |
|  | subtotal |  |  |  | 0.220（0.070-0.417） |
| DCR | Ghosal 2011 | Imatinib + cisplatin | 21 | 28 | 0.750（0.566-0.873） |
|  | Hitre 2013 | Cetuximab + chemotherapy | 16 | 21 | 0.762(0.549-0.894) |
|  | subtotal |  |  |  | 0.755（0.622-0.869） |

**Table S3 The summary of grade 3 or higher adverse events**

| **Study** | **Drug** | | **Total** | **Events** | | |
| --- | --- | --- | --- | --- | --- | --- |
| Hotte 2004 | Imatinib | | 16 | 15 | | |
| Ching-Hung Lin2005 | Imatinib | | 5 | 3 | | |
| Ghosal 2011 | Imatinib+Cisplatin | | 28 | 6 | | |
| Chau, NG 2012 | Sunitinib | | 13 | 16 | | |
| Hitre 2013 | Cetuximab | | 21 | 19 | | |
| Kim 2014 | Everolimus | | 34 | 4 | | |
| Thomson 2014 | Sorafenib | | 23 | 23 | | |
| Keam 2015 | Dovitinib | | 32 | 45 | | |
| Ho,A.L.2015 | AKT inhibitor (MK-2206) | | 16 | 8 | | |
| Ho, A. L. 2016 | Axitinib | | 33 | 16 | | |
| Goncalves 2017 | Vorinostat | | 30 | 8 | | |
| Locati2020 | Lenvatinib | | 28 | 21 | | |
| Kang 2021 | Vorinostat | | 60 | 17 | | |
| Guopei Zhu 2021 | Apatinib | | 68 | 24 | | |
| Hanna2021 | ATRA | | 18 | 4 | | |
| Ning Su 2022 | Apatinib | | 19 | 1 | | |
| **Table S4** The incidence rate of the most common all-grade adverse events in MKIs | | | | | |  |
|  |  |  |  |  |  |  |
| AEs | | Any grade | | | |  |
|  |  | ES, % (95% CI) | | | I2, % |  |
| Fatigue | | 52.5 (30.2-74.3) | | | 93.7 |  |
| Hypertension | | 46.1 (24.9-67.9) | | | 94.3 |  |
| Nausea | | 43.4 (24.4-63.4) | | | 90.3 |  |
| Hand–foot syndrome | | 36.8 (19.2-56.3) | | | 88 |  |
| Diarrhea | | 36.1 (18.8-55.2) | | | 91.9 |  |
| Anorexia | | 34.6 (22.1-48.3) | | | 75.7 |  |
| Weight loss | | 34.3 (16.4-54.8) | | | 90.3 |  |
| Headache | | 29.0 (12.8-48.3) | | | 88.2 |  |
| Vomiting | | 25.7 (14.8-38.1) | | | 59.9 |  |
| Proteinuria | | 25.4 (17.2-34.6) | | | 62.1 |  |
| Rash | | 20.8 (7.4-38.1) | | | 88.3 |  |
| Thrombocytopenia | | 11.1 (4.1-20.4) | | | 50.8 |  |
| Anemia | | 9.2 (5.3-13.8) | | | 13.0 |  |

Supplementary Figure Caption

**Supplemental Figure S1** Sensitivity analysis for DCR in R/M ACC.

**Supplemental Figure S2** Sensitivity analysis for ORR in R/M ACC.

**Supplemental Figure S3** Publication biases for DCR in R/M ACC.

**Supplemental Figure S4** Publication biases for ORR in R/M ACC.
