# Supplementary figures and images for: The effectiveness of targeted therapy for recurrence or metastasis adenoid cystic carcinoma: a systematic review and meta-analysis
Source: Ann Med. 2024 Sep 11;56(1):2399867. doi: 10.1080/07853890.2024.2399867 (PMC11391875; doi:10.1080/07853890.2024.2399867)

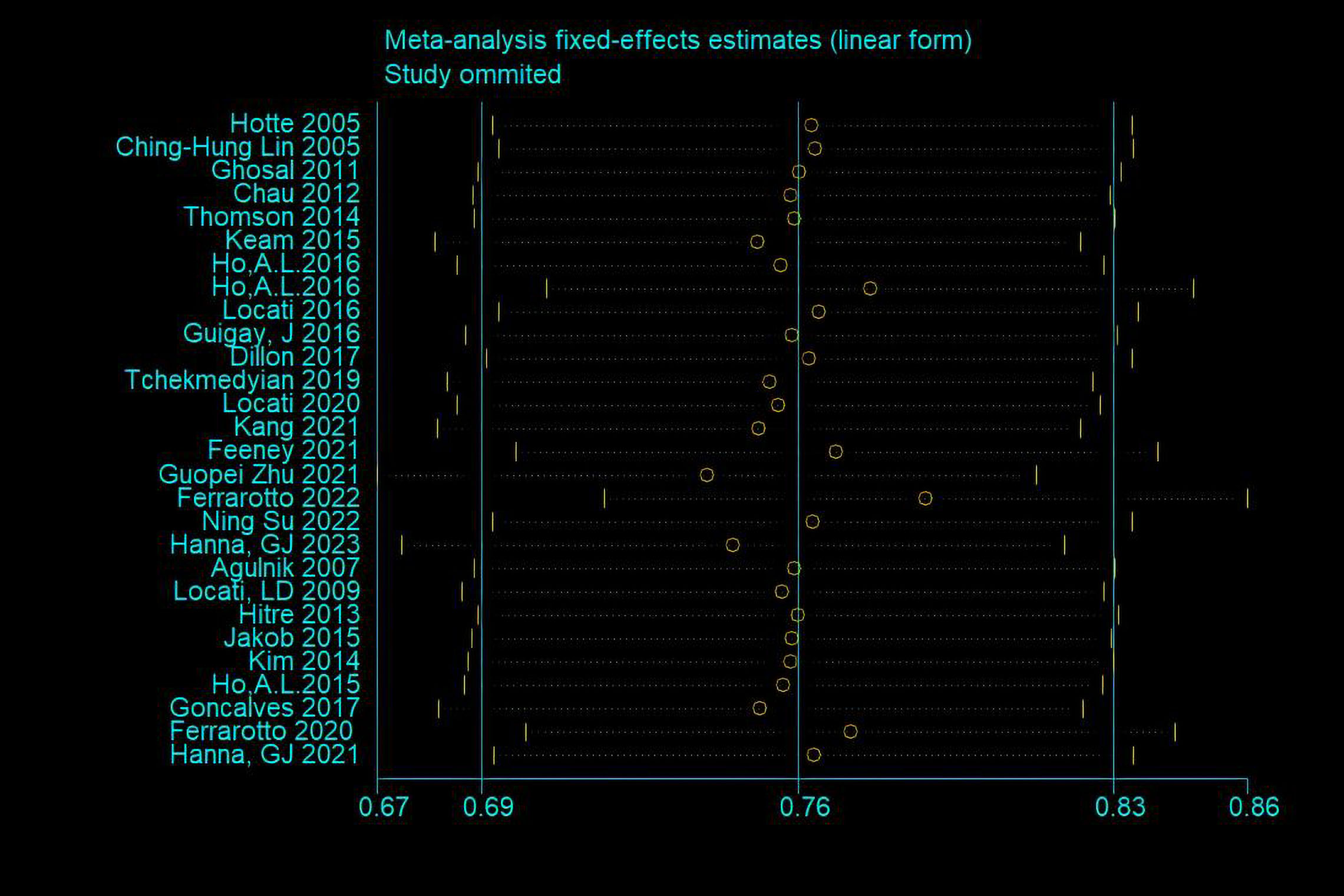

Supplement: Supplemental Material [file IANN_A_2399867_SM5405.zip › suppl_data/Supplementary Figure S1 color figure.tif]

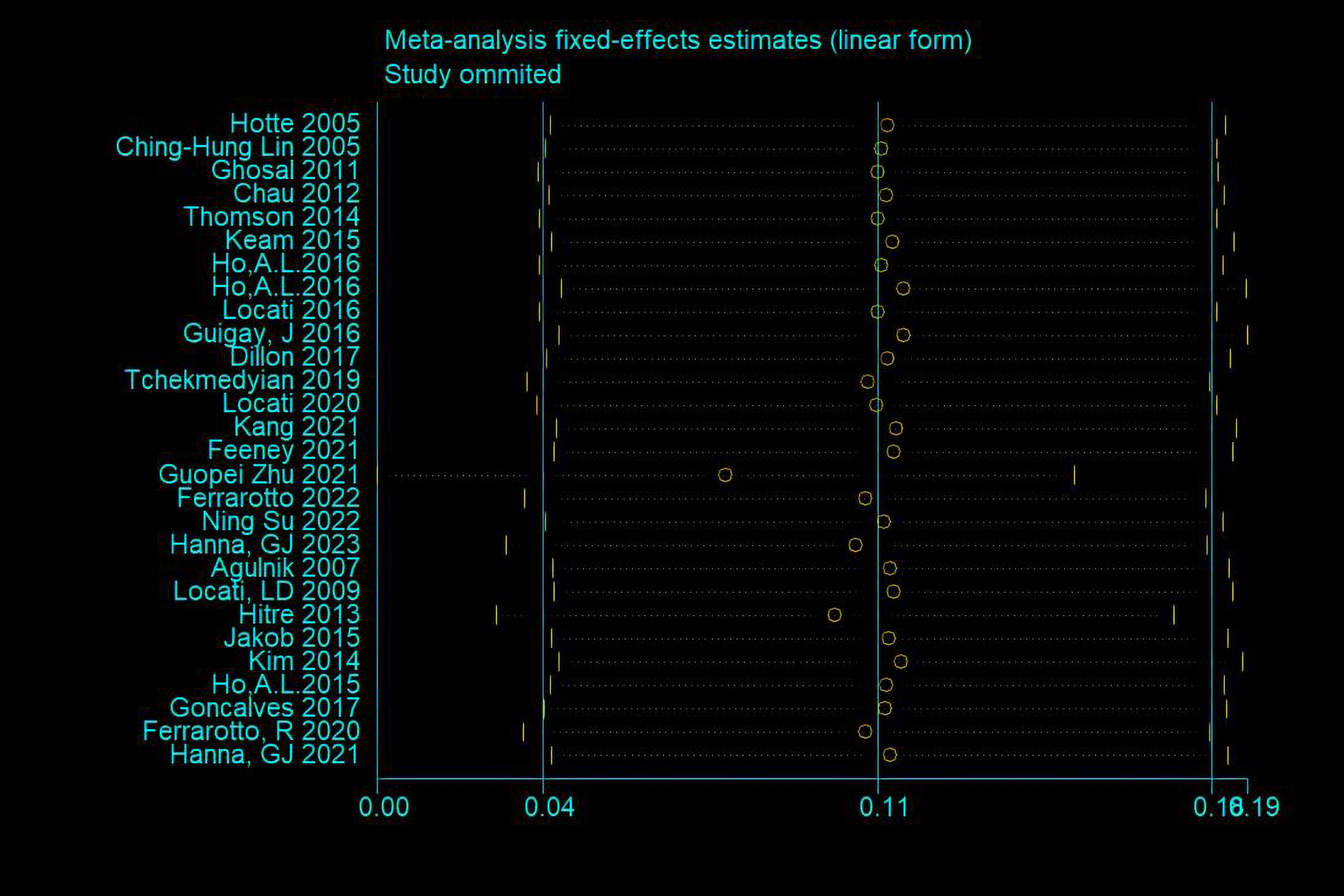

Supplement: Supplemental Material [file IANN_A_2399867_SM5405.zip › suppl_data/Supplementary Figure S2 color figure.tif]

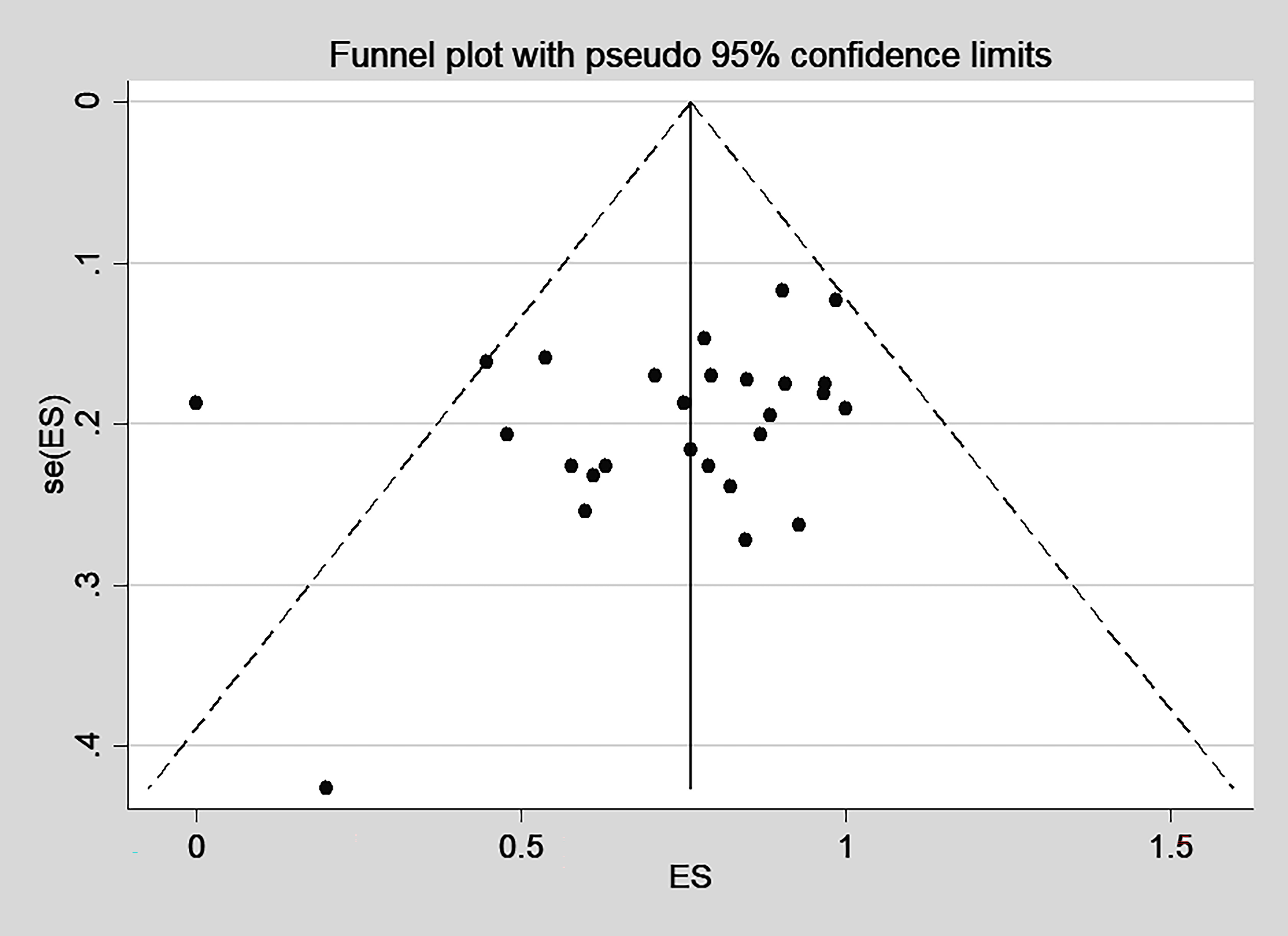

Supplement: Supplemental Material [file IANN_A_2399867_SM5405.zip › suppl_data/Supplementary Figure S3-1.tif]

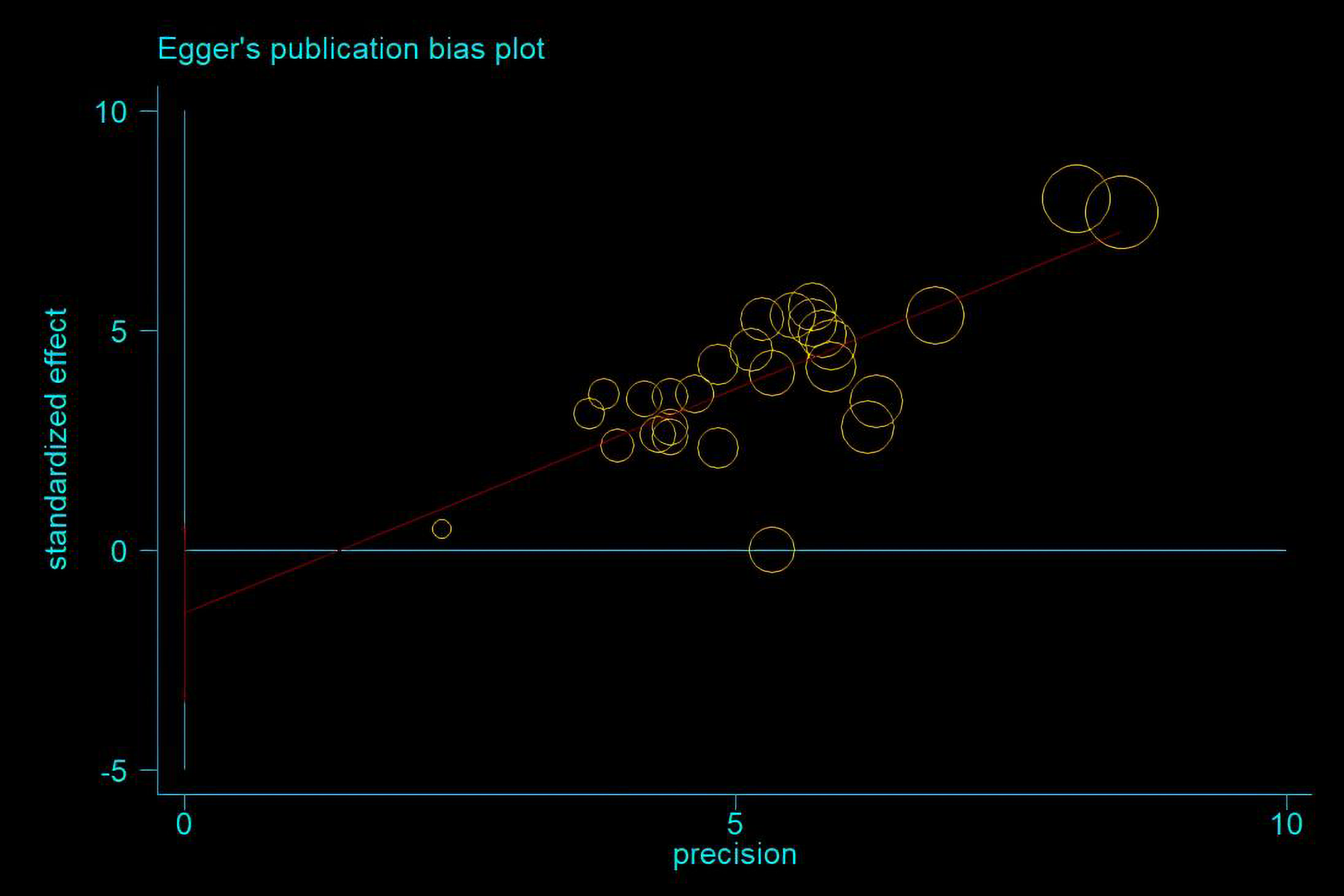

Supplement: Supplemental Material [file IANN_A_2399867_SM5405.zip › suppl_data/Supplementary Figure S3-2 color figure.tif]

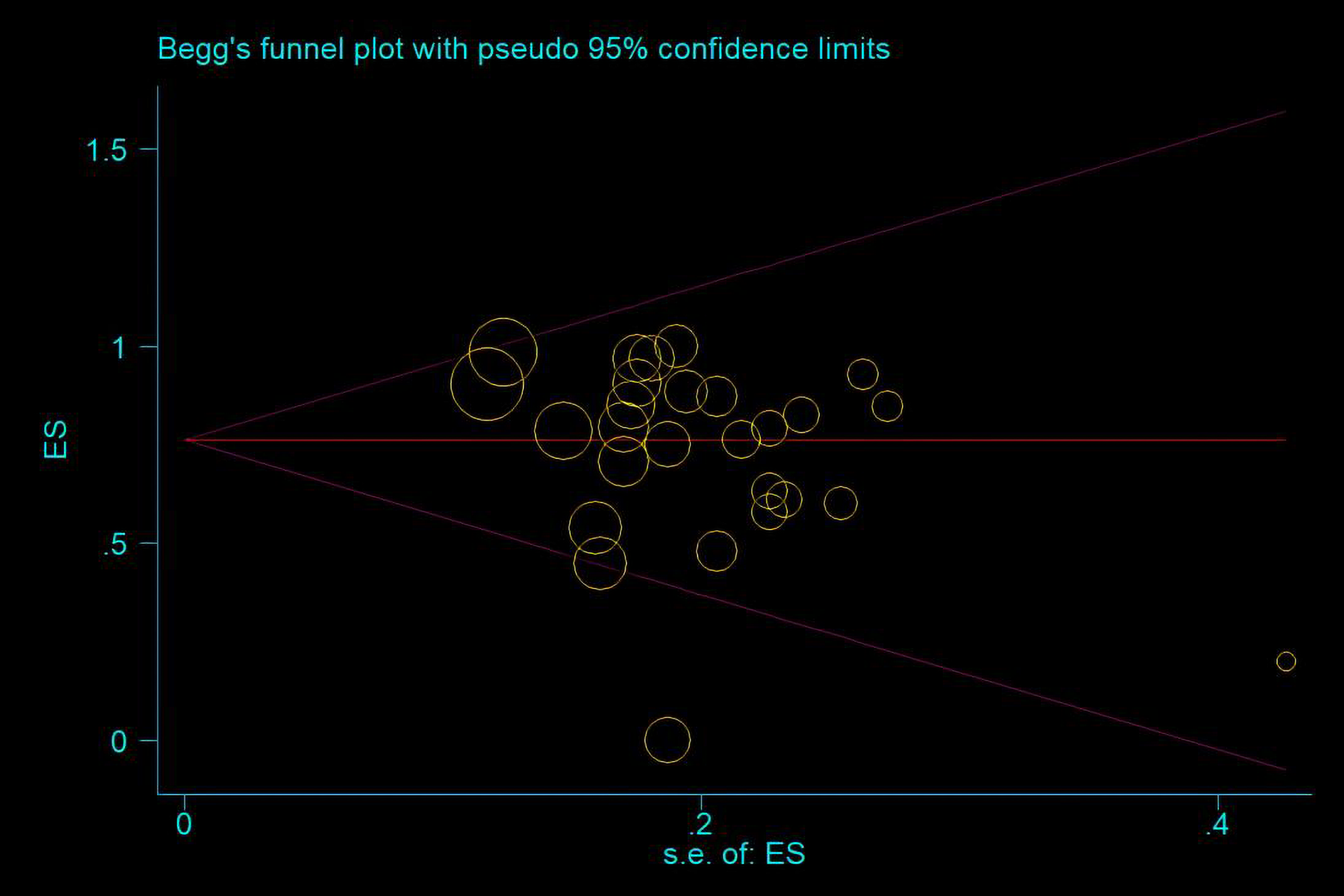

Supplement: Supplemental Material [file IANN_A_2399867_SM5405.zip › suppl_data/Supplementary Figure S3-3 color figure.tif]

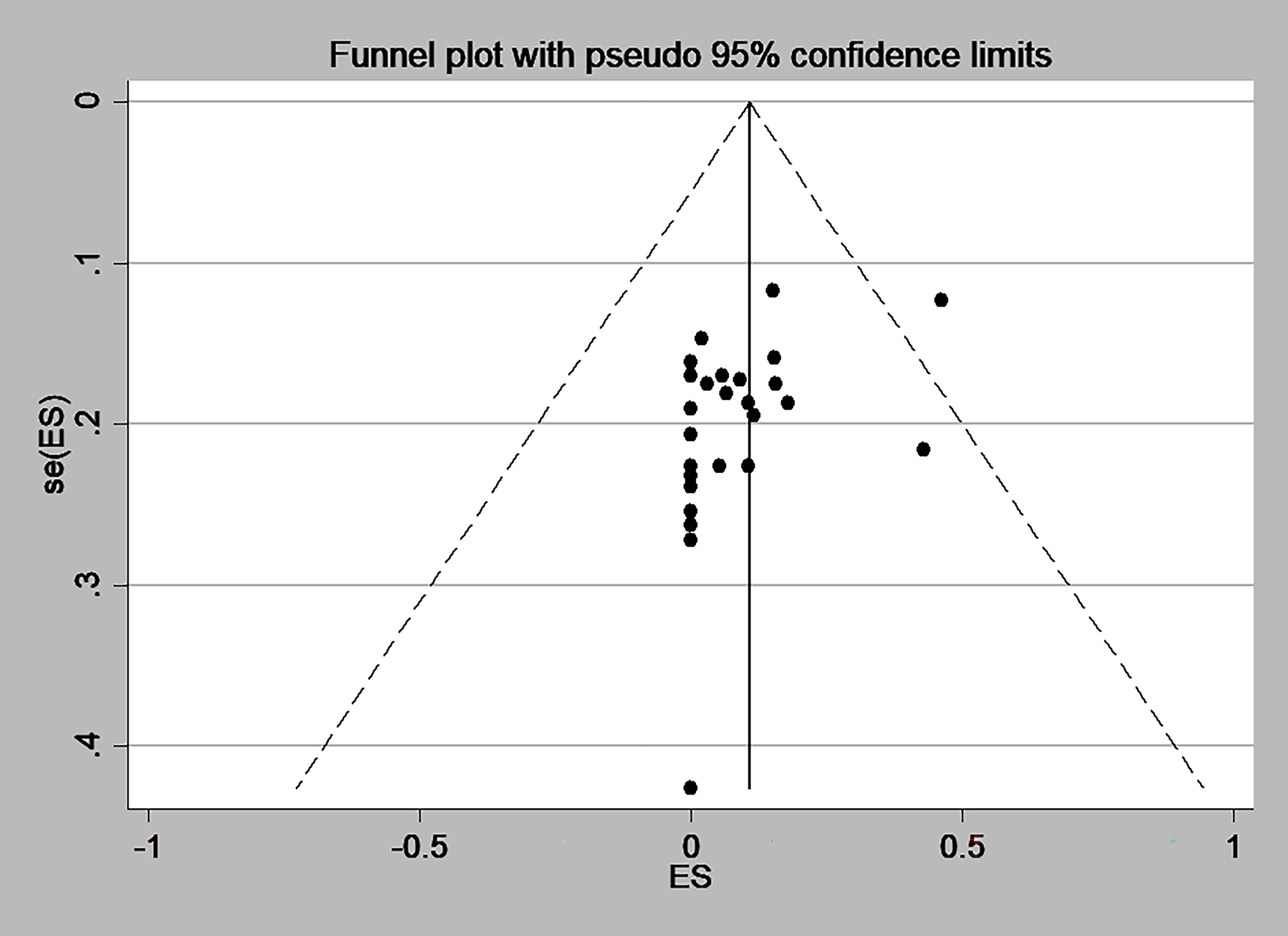

Supplement: Supplemental Material [file IANN_A_2399867_SM5405.zip › suppl_data/Supplementary Figure S4-1.tif]

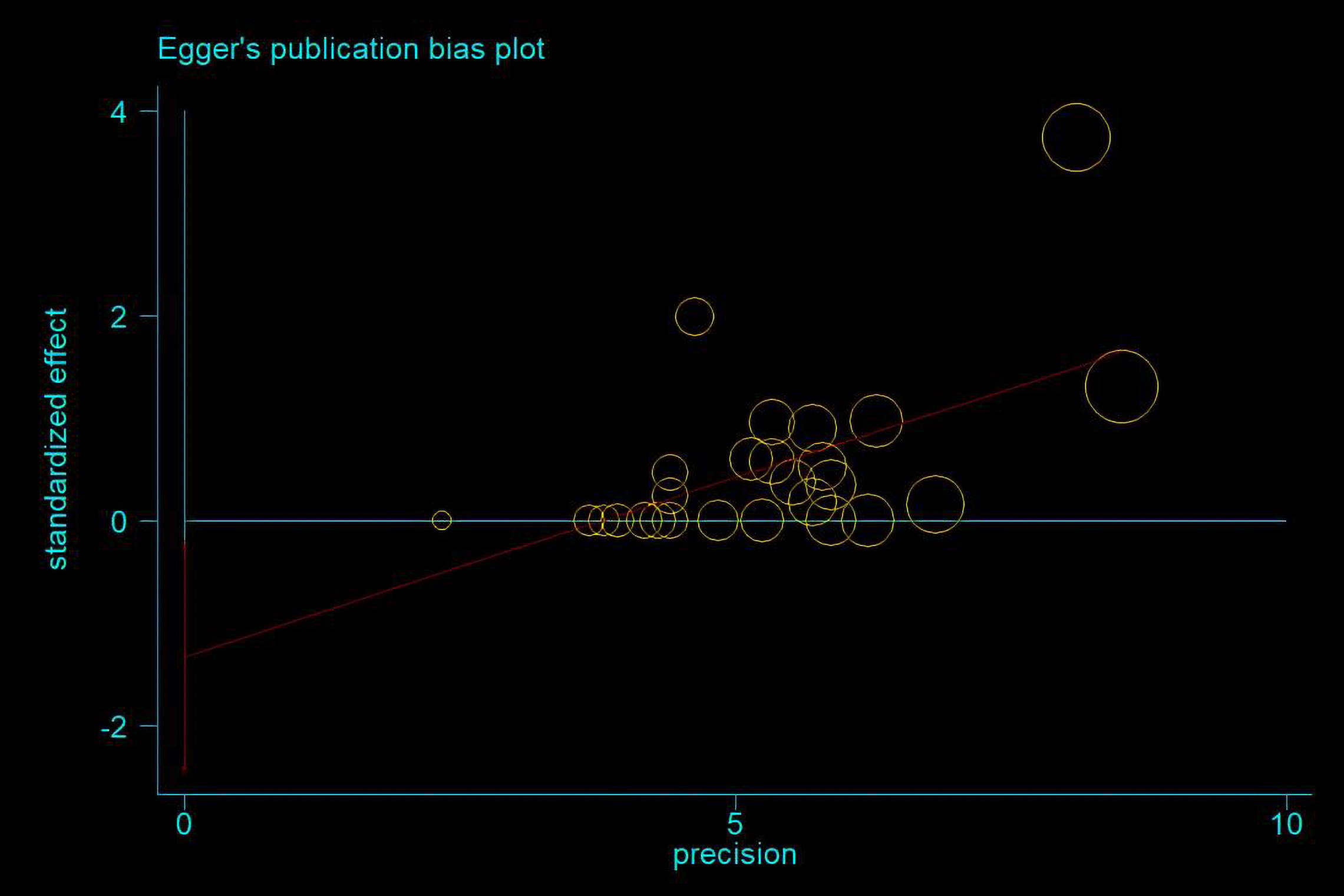

Supplement: Supplemental Material [file IANN_A_2399867_SM5405.zip › suppl_data/Supplementary Figure S4-2 color figure.tif]

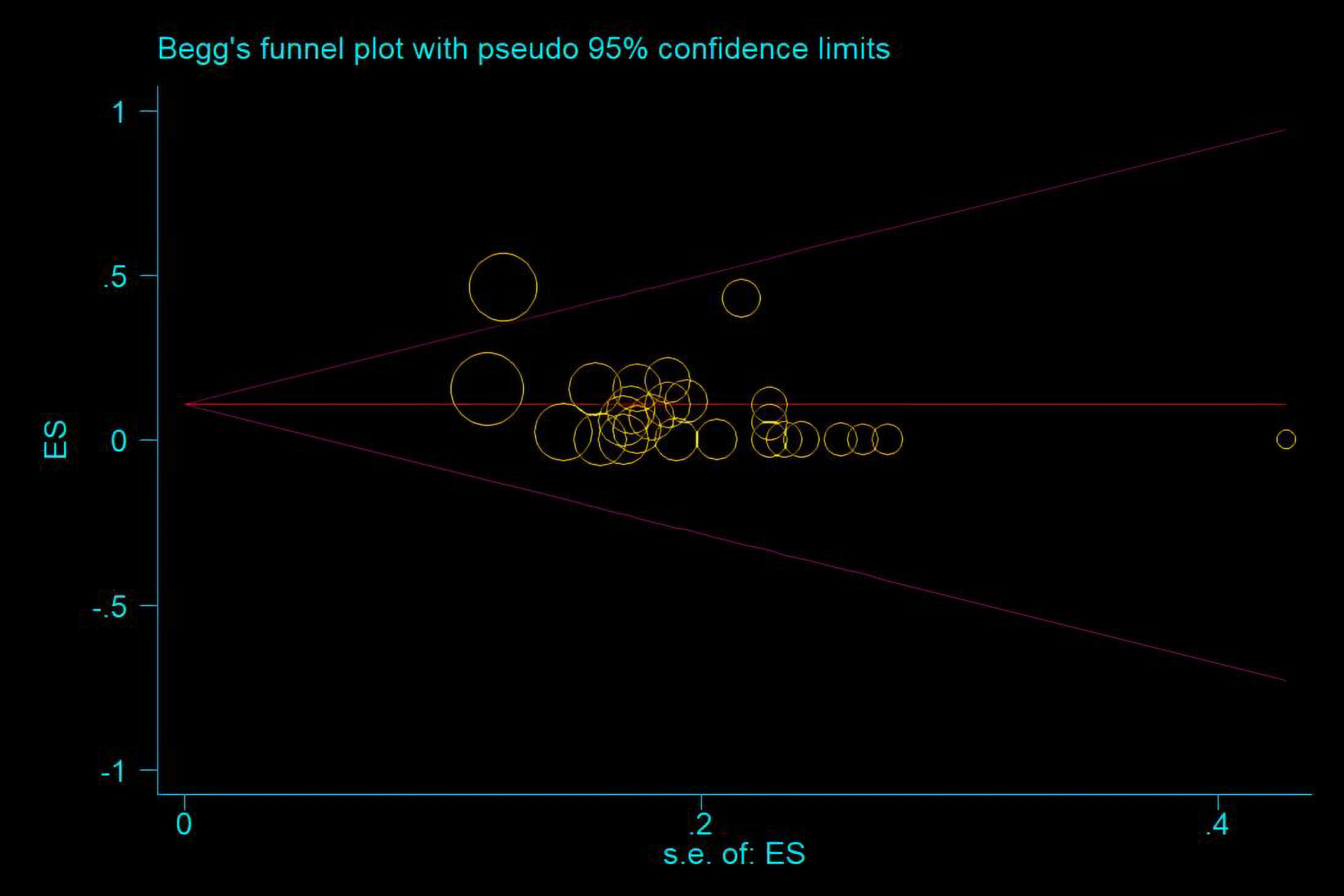

Supplement: Supplemental Material [file IANN_A_2399867_SM5405.zip › suppl_data/Supplementary Figure S4-3 color figure.tif]
